# Sun Exposure Behaviour and Vitamin D in South Asian Compared with White Caucasian Adolescents: Prospective Cohort Study

**DOI:** 10.3390/nu18152443

**Published:** 2026-07-27

**Authors:** Elizabeth J. Marjanovic, Mark D. Farrar, Richard Kift, Mohamed Z. Mughal, Ann R. Webb, Lesley E. Rhodes

**Affiliations:** 1 Centre for Dermatology Research, Division of Musculoskeletal and Dermatological Sciences, School of Biological Sciences, Faculty of Biology, Medicine and Health, NIHR Manchester Biomedical Research Centre, The University of Manchester, Manchester M13 9PL, UK; elizabeth.marjanovic@manchester.ac.uk (E.J.M.); mark.farrar@manchester.ac.uk (M.D.F.); 2 Photobiology Unit, Dermatology Centre, Salford Royal Hospital, Northern Care Alliance NHS Foundation Trust, Manchester Academic Health Science Centre, Salford M6 8HD, UK; 3 Department of Earth and Environmental Sciences, Faculty of Science and Engineering, The University of Manchester, Manchester M13 9PL, UK; richard.kift@manchester.ac.uk (R.K.); ann.webb@manchester.ac.uk (A.R.W.); 4 Department of Pediatric Endocrinology, Al Jalila Children’s Hospital, Dubai Health, Al Jaddaf, Dubai P.O. Box 7662, United Arab Emirates; mohamed.mughal@dubaihealth.ae

**Keywords:** adolescents, Vitamin D, skin type, sunlight, ultraviolet radiation, guidance

## Abstract

**Background:** Sun exposure guidance advises minimisation to reduce skin cancer risk, but advice is geared for white-skinned people. Melanin reduces skin synthesis of vitamin D from solar ultraviolet radiation (UVR), and adolescence is a key life-stage for bone mass acquisition. **Methods:** This prospective cohort study investigated year-round vitamin D status and its determinants in 143 South Asian adolescents (12–15 y; brown skin) living in Greater Manchester, UK. Serum 25OHD, personal UVR doses, time outdoors, and photoprotective measures were assessed across four seasons, and the findings were compared with those of 131 white Caucasian adolescents studied using identical protocols. **Results:** Severe vitamin D deficiency (25OHD < 25 nmol/L) was prevalent in South Asians, affecting 79% of girls and 69% of boys in winter, persisting year-round in 59% of girls and 23% of boys, and accompanied by winter PTH levels > the upper reference range in 59% of boys and 45% of girls. Personal UVR doses and time outdoors were lower on summer weekend days (boys 53 min, girls 60 min) vs. schooldays (boys 105 min, girls 108 min) in South Asians (*p* < 0.01). The findings contrasted with those in white Caucasians, where time outdoors was similar on summer weekend days and schooldays (weekend days: boys 131 min, girls 101 min, *p* < 0.01 vs. South Asians). Only 26% of South Asians vs. 86% of white Caucasians took a holiday abroad during the year, and days abroad significantly contributed to vitamin D status. Both groups had low oral vitamin D intake. **Conclusions:** Raising vitamin D status is crucial to prevent secondary hyperparathyroidism in South Asian adolescents and skin type-related sun exposure guidance is required.

## 1. Introduction

Vitamin D is a unique nutrient, derived both from skin synthesis following solar ultraviolet B (UVB) radiation exposure, and orally through diet and supplements. Melanin attenuates skin penetration of UVB and hence reduces this major source of vitamin D, whilst few foodstuffs contain more than modest amounts of vitamin D. Vitamin D is essential for normal bone formation and mineralization in children, including in older childhood, with ~40% of adult peak bone mass acquired in the years around puberty [1,2]. Vitamin D deficiency in adolescence can lead to the severe bone disorder of osteomalacia, and may impair bone health in later life, increasing the risk of fracture and osteoporosis [3,4]. Low vitamin D status is also associated with increased risk of respiratory infection [5], autoimmune disease, and a range of other conditions [6], although causality is not proven [7].

There is a paucity of data on longitudinal vitamin D status in South Asian children, with studies that have been reported being cross-sectional or in only one season. A UK study (*n* = 618) showed serum 25-hydroxyvitamin D (25OHD) < 25 nmol/L (severe deficiency) in 20–34% of UK-born 2-year old South Asian infants, with blood samples gathered in a single season (autumn) [8]. Another UK study in children aged 4–18 reported that 85% of their non-white children’s population had 25OHD < 50 nmol/L (deficiency/insufficiency) [9]. However, only 51 South Asian adolescents were included, and blood was sampled once. Internationally, the influence of ethnicity/skin type on 25OHD is supported by the NHANES study, which found that in *n* = 903 USA non-Hispanic Black children, 43% of boys and 59% of girls aged 13–21 years had 25OHD < 37.5 nmol/L [10], and a study by Weng et al. [11] reported that of *n* = 141 African Americans aged 6–21 years, 133 (94%) had winter 25OHD levels < 75 nmol/L. Additionally, Zhang et al. [12] reported that 60% of Chinese children (*n* = 110 of 185) aged 12–17 years had 25OHD < 37.5 nmol/L across all seasons.

Little is known regarding the sun exposure behaviour of South Asian children living in higher latitude countries. Guidance on sun exposure and sun protection for younger age groups is principally geared for white children, leading to uncertainty for parents and children of darker skin types on measures and behaviours to adopt. Sun exposure guidance encourages staying in the shade, covering up with clothing, and regular use of sunscreen to reduce skin cancer risk, while recognising that brief sun exposures are advisable to assist vitamin D status [13,14]. However, skin cancer risk is much lower in South Asians than in the white population, while those living in higher latitude countries have high risk of vitamin D deficiency and insufficiency [15,16,17].

The aims of this study were to define (i) the year-round vitamin D status and (ii) the contributions of sunlight exposure and diet to vitamin D status, including detailed photobiological assessment, in adolescents of South Asian ethnicity (skin type V). Additionally, (iii) to contextualise the findings, comparisons were made with data from a cohort of white Caucasian adolescents (skin type I–IV) [4] living at the same location and studied throughout the seasons under identical protocols.

## 2. Materials and Methods

### 2.1. Study Design and Participants

This was a single-centre cohort study in healthy, ambulant South Asian (Fitzpatrick skin type V, [18]) adolescents. The children were recruited through four fee-paying (private) schools and three free (state) schools in Greater Manchester, UK (53.5° N). The study was performed between January and September 2013. In winter (January), spring (April), summer (June), and autumn (September), blood samples were taken for 25OHD, personal sunlight exposure and ambient UVB levels were evaluated, and dietary vitamin D intake was estimated. Inclusion criteria were girls and boys aged 12–15 years of South Asian ethnicity with sun-reactive skin type V (brown skin). Exclusion criteria were history of photosensitivity or being non-ambulant. Participants were asked to continue their usual practice of vitamin D supplementation during the study period. Ethical approval was obtained (University of Manchester Research Ethics Committee, Project Ref 11320) and participants gave written informed consent. Data from the South Asian adolescents was compared with data from a study performed between January and September 2011, using an identical protocol at the same study site, in white Caucasian adolescents, sun-reactive skin types I–IV [18], aged 12–15 years, with exclusion criteria of history of photosensitivity or being non-ambulant [4]. The study was performed according to the Declaration of Helsinki principles.

### 2.2. Personal Ultraviolet Radiation Exposure

Adolescents wore polysulphone film badge UVR dosimeters [4,16] for 1 week in each season. These were attached to the outer clothing of upper chest/anterior shoulder, with one badge for the 5 weekdays and a separate badge for the weekend. Dosimeters recorded total personal erythemal UVR dose, reflecting their UVB exposure. Badges were included in analyses unless unworn (determined from information from subject or absent pinhole in badge holder).

### 2.3. Time Spent Outdoors

Adolescents concurrently completed sun exposure diaries for 1 week in each season, encompassing both schooldays and weekends. Diary days were excluded from analyses if not completed. These detailed the time outdoors (in 15 min blocks), weather conditions, clothing worn, and the use of dedicated sunscreen products and sun protection factor-containing face products [4]. Adolescents also recorded the number of days spent abroad during the study year and the location of the visit. Sunny holidays were categorised as visits abroad to countries with a more southerly latitude than the UK (latitudes south of 51° N).

### 2.4. Dietary Vitamin D Intake

Oral vitamin D intake was estimated through daily dietary log recordings: vitamin D supplements; vitamin D-fortified foods; oily fish; butter, margarine and other spreads; milk; eggs; cheese; and red meat [19,20]. Diary days were excluded from analyses if not completed.

### 2.5. Serum 25-Hydroxyvitamin D and Biochemical Analyses

Blood was sampled the week following the badge recording of UVR exposure and the completion of sun exposure diaries. Serum was stored at −20 °C until study completion. Serum concentrations of 25OHD were determined by liquid chromatography tandem mass spectrometry (ABS 5500 LC-MS/MS, Chromsystems 25OHD kit, ABSciex, Framingham, MA, USA), following the manufacturers’ instructions (intra- and inter-assay coefficients of variation of 3.7% and 4.8%, respectively). The assay laboratory (Manchester Royal Infirmary) had Clinical Pathology Accreditation UK (No. 0865) and was certified proficient by the Vitamin D Quality Assurance Scheme (DEQAS). Serum parathyroid hormone (PTH) was assessed in samples taken in winter and autumn in order to investigate whether secondary hyperparathyroidism, resulting from low serum 25OHD, was influenced by vitamin D status at the end of summer (early autumn), when 25OHD levels would be at their peak, compared with the end of winter, when levels would be at their lowest. PTH was measured following the manufacturer’s instructions (OCTEIA immunoenzymometric assay, Immunodiagnostic Systems Ltd., Boldon, UK), with a sensitivity of 0.057 pg/mL (intra- and inter-assay coefficients of variation of 4% and 6%, respectively). Serum biochemistry was assessed at baseline including calcium and renal and liver function. Reference ranges: PTH (11–18 y) 15.09–67.92 pg/mL; alkaline phosphatase (ALP) 10–12 y 129–417 U/L, 13–14 y M116–468: F57–254 U/L, and 15–16 y M82–331: F50–117 U/L; phosphate (12–16 y) 0.95–1.5 mmol/L.

### 2.6. Statistical Analysis

A sample size of 100 was required to accurately estimate population standard deviation for outcomes [21]; recruitment of 125 children allowed for 20% drop-out. This sample size gives 80% power at 5% significance to detect correlations greater than 0.3. Multiple linear regression explored associations between late summer peak 25OHD and demographic and behavioural characteristics, including sunlight exposure and dietary vitamin D intake; models first assessed how much 25OHD variation the demographic factors could explain, following which behavioural factors were added. Sun exposure, 25OHD, and body mass index data were log-transformed to linearize relationships and stabilize variance. Proportion of explained variance was quantified by reduction in R^2^ as behavioural variables were removed from the model. To reduce “overfitting,” variables were selected based on substantive interest and previous multivariable analyses of other cohorts [4]. Analyses were performed on an available-case basis and assumed no systematic differences between subjects with complete and missing data, beyond those accounted for by factors included in the model.

Post hoc comparisons were made between males vs. females and South Asian vs. white adolescents using the Mann–Whitney test, and within-season weekday vs. weekend UVR exposure and time outdoors were compared by the Wilcoxon signed-rank test, without adjustment for multiple testing.

## 3. Results

### 3.1. Participant Characteristics

The study population comprised 143 South Asian adolescents. Three withdrew after the first data collection period and two withdrew following the second. In total, 138 children provided blood samples (88 in all four seasons, 34 in three seasons, 10 in two seasons, and six in one season). The total number of adolescents that completed the badge-wearing exercise was 113 (82%) for weekdays and 109 (79%) for weekends, completion of sun exposure diaries was 84 to 103 (60 to 74%), and completion of dietary logs was 83 to 101 (60 to73%) across seasons. Baseline characteristics are shown in Table 1, which also includes a comparison with the data from 131 white Caucasian adolescents undergoing the same study protocols [4]. The following sections show the study parameters as assessed in every season, i.e., winter (baseline), spring, autumn, and winter.

### 3.2. Seasonal Serum 25-Hydroxyvitamin D

**Figure 1:**
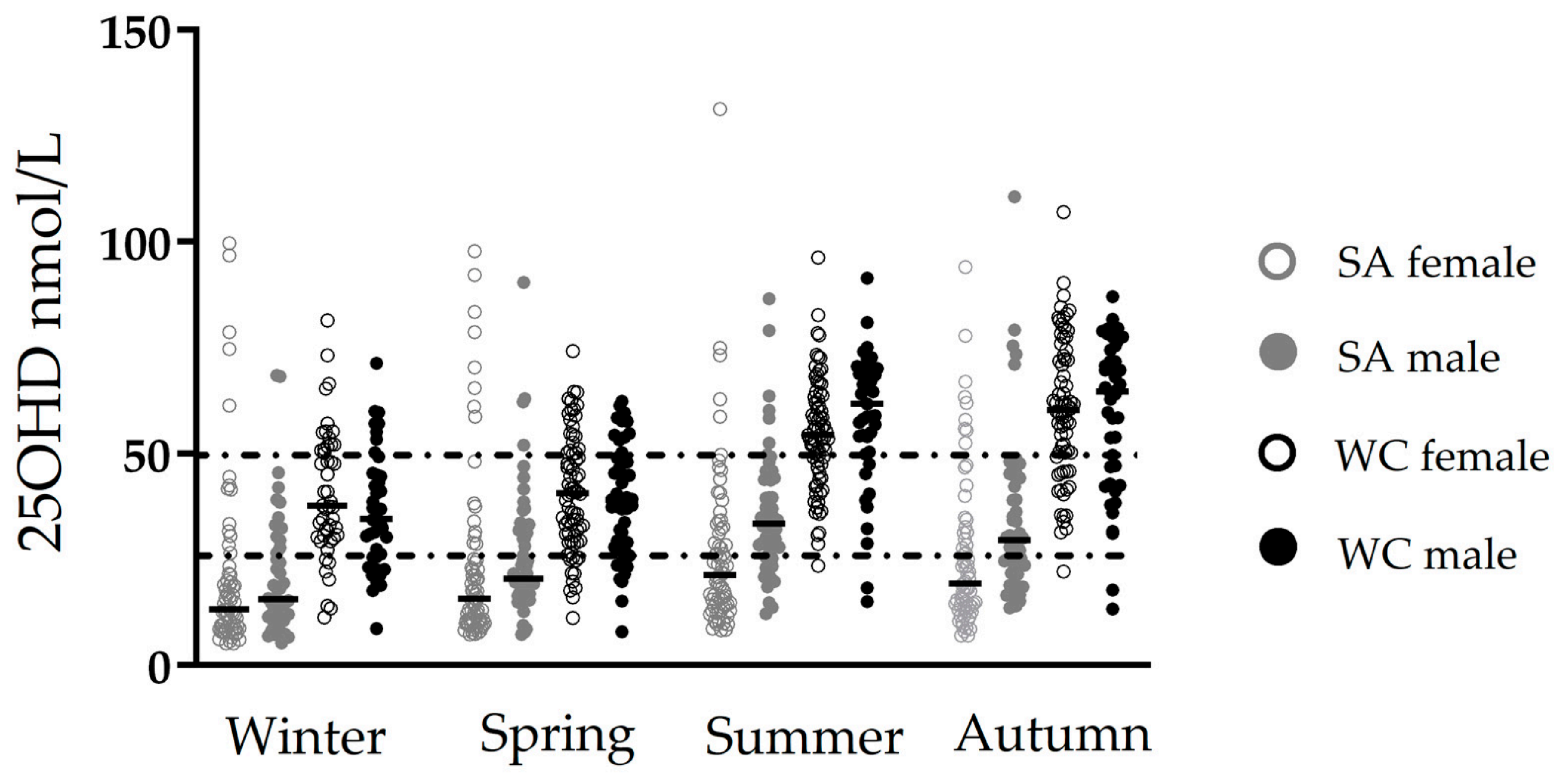
Seasonal 25OHD values in South Asian (SA) and white Caucasian (WC) adolescents. Dashed lines show deficient (25 nmol/L) and sufficient (50 nmol/L) levels. Bold lines show median values for each group. SA: winter n = 110, spring n = 131, summer n = 123, and autumn n = 116; WC: winter n = 90, spring n = 120, summer n = 118, and autumn n = 115.

There was only a weak seasonal pattern in vitamin D status in the South Asian adolescents (Figure 1). The peak median 25OHD occurred in summer with 21.3 (range of 13.9 to 32.8) nmol/L in females and 33.4 (26.6 to 44.4) nmol/L in males, and the trough occurred in winter with values of just 13.2 (8.7 to 21.6) nmol/L in females and 15.5 (11.2 to 28.2) nmol/L in males. The South Asian adolescents had significantly (*p* < 0.001) lower 25OHD levels than the white Caucasian adolescents at both the peak (27.8 (16.8 to 39.5) vs. 61.7 (46.9 to 73.0) nmol/L) and winter trough periods (14.8 (9.4 to 25.4) vs. 36.9 (29.3 to 50.1) nmol/L).

**Figure 2:**
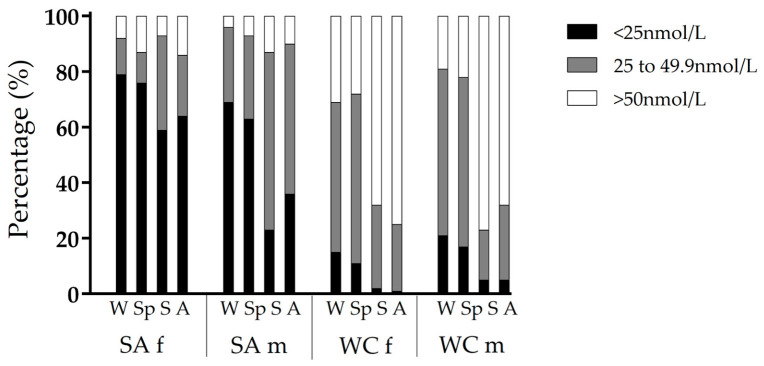
Percentage of South Asian (SA) and white Caucasian (WC) male (m) and female (f) adolescents who were vitamin D sufficient (25OHD ≥ 50 nmol/L), insufficient (25–50 nmol/L), and deficient (<25 nmol/L) in winter (W), spring (Sp), summer (S), and autumn (A).

The percentage of participants who were severely vitamin D deficient (25OHD < 25 nmol/L) is shown (Figure 2). In winter, 79% of female and 69% of male South Asian adolescents were deficient. In summer, this percentage reduced to 59% of females and 23% of males, such that the majority of South Asian females remained deficient year-round. In total, 110 South Asian adolescents were vitamin D deficient in at least one season. Of those South Asian adolescents who had values for all seasons, 32 were deficient and only three were sufficient all year round. In white Caucasian adolescents, deficiency was highest in winter, although it was much less prevalent than in South Asian adolescents, with 15% of females and 21% of males having 25OHD < 25 nmol/L.

### 3.3. Parathyroid Hormone

**Figure 3:**
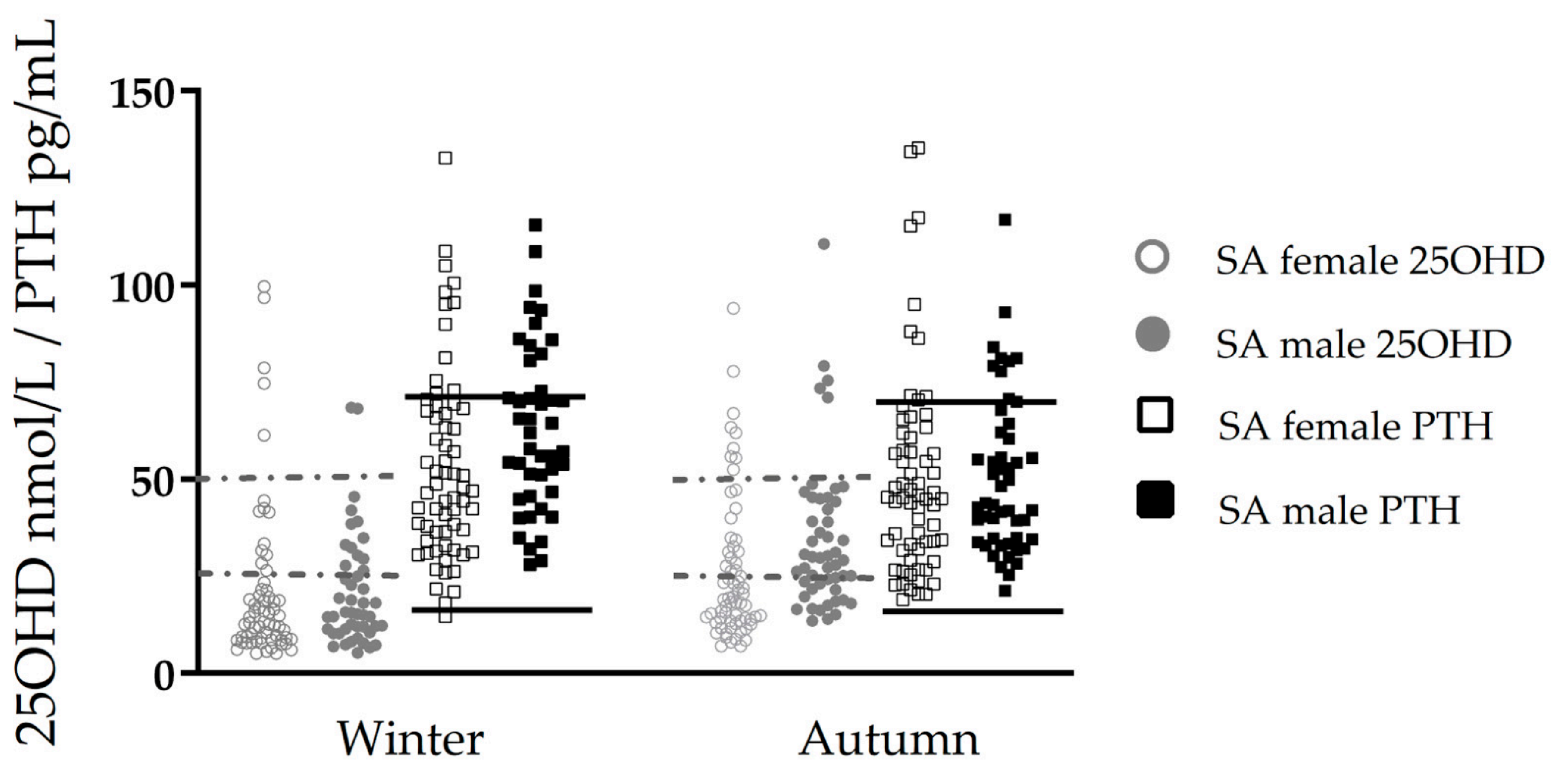
PTH (pg/mL) and 25OHD (nmol/L) levels recorded in South Asian (SA) females and males in winter and autumn (winter n = 151, autumn n = 151). Dashed lines show deficient (25 nmol/L) and sufficient (50 nmol/L) levels for 25OHD and bold lines show deficient (15.1 pg/mL) and sufficient (67.9 pg/mL) levels for PTH.

The percentages of South Asian females, South Asian males, white Caucasian females, and white Caucasian males, respectively, whose PTH levels were > 67.92 pg/mL in winter were 45%, 59%, 50%, and 29%, and in autumn the values were 38%, 38%, 15%, and 22% (Figure 3). The median (IQR) PTH levels were significantly higher in South Asian females relative to white Caucasian females in both winter and autumn: 47.9 (35.2 to 68.6) vs. 39 (32.3 to 46) pg/mL (*p* = 0.01) in winter and 45.2 (31.8 to 61.3) vs. 33.0 (28 to 45.3) pg/mL (*p* < 0.001) in autumn. The median PTH levels were significantly higher in South Asian males relative to white Caucasian males in winter, 63.2 (46.5 to 82.9) vs. 45 (36.5 to 58.5) pg/mL (*p* < 0.001), although not in autumn, 43.6 (34.3 to 62.6) vs. 43.5 (39.3 to 54) pg/mL (*p* = 0.93).

The percentages of South Asian females, South Asian males, white Caucasian females, and white Caucasian males, respectively, who had both high PTH (>67.92 pg/mL) and very low 25OHD (<25 nmol/L) in winter were 15%, 27%, 3%, and 6%. PTH was significantly negatively correlated with 25OHD in South Asian males (r = −0.43, *p* < 0.001), although not females (r = −0.24, *p* = 0.06), in winter, and in South Asian females (r = −0.32, *p* = 0.01), although not males (r = −0.15, *p* = 0.32), in autumn. No significant relationship was observed between PTH and 25OHD in white Caucasian males and females in either winter or autumn.

### 3.4. Calcium, Phosphate, and Alkaline Phosphatase

In winter, median (IQR) serum calcium levels were 2.45 (2.38 to 2.51), 2.44 (2.37 to 2.49), 2.45 (2.41 to 2.49), and 2.42 (2.39 to 2.46) mmol/L, respectively, in South Asian females, South Asian males, white Caucasian females, and white Caucasian males. Winter phosphate winter levels were 1.24 (1.13 to 1.31), 1.51 (1.37 to 1.62), 1.35 (1.22 to 1.46), and 1.48 (1.35 to 1.61) mmol/L, respectively, in South Asian females, South Asian males, white Caucasian females, and white Caucasian males. Median (IQR) winter serum ALP levels were 86 (71 to 105), 208 (167 to 265), 148 (98 to 198), and 202 (162 to 254) U/L, respectively, in South Asian females, South Asian males, white Caucasian females, and white Caucasian males. The percentages with serum ALP above the reference range for age and gender were 15%, 2%, 6%, and 2%, respectively, in South Asian females, South Asian males, white Caucasian females, and white Caucasian males.

### 3.5. Dietary Vitamin D Intake

In the South Asian adolescents, total oral intake of vitamin D from food and supplements was very low in all seasons: median (IQR): winter 1.52 (0.92 to 2.74) μg/day, spring 1.48 (0.86 to 2.68) μg/day, summer 1.50 (0.66 to 2.54) μg/day, and autumn 1.36 (0.56 to 2.28) μg/day. The overall, year-round median (IQR) intake was 1.76 (1.03 to 3.29) μg/day in boys and 1.39 (0.78 to 2.21) μg/day in girls. Twenty-eight (23%) South Asian adolescents reported taking supplements containing vitamin D in at least one season and only five (4%) took supplements year-round, with the amount of vitamin D obtained from supplements ranging from 0.7 to 20 μg/day. White Caucasian adolescents’ oral intake of vitamin D was also low: year-round median (IQR): 1.97 (1.11 to 3.22) μg/day in females and 1.73 (1.14 to 2.69) μg/day in males. Twenty-seven (21%) white Caucasian adolescents reported taking supplements containing vitamin D in at least one season but only five (4%) adolescents reported taking supplements year-round. Intake of vitamin D was significantly different between the South Asian females and white Caucasian females, who had the lowest and highest intakes, respectively (*p* = 0.014).

### 3.6. Personal Ultraviolet Radiation Exposure

**Figure 4:**
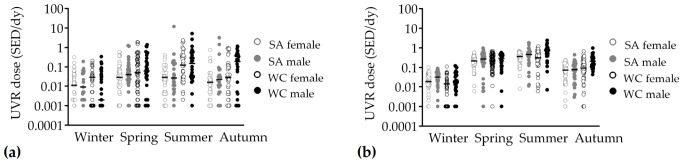
UVR dose (standard erythemal dose, SED) per day in South Asian (SA) and white Caucasian (WC) adolescents on (**a**) weekdays and (**b**) weekends (median shown as bold lines). SA: winter *n* = 86, spring *n* = 104, summer *n* = 83, and autumn *n* = 88; WC: winter *n* = 96; spring *n* = 101; summer *n* = 86; and autumn *n* = 78.

Personal UVR exposure was quantified using polysulphone badges (Figure 4a,b). Median daily UVR dose gained by South Asian adolescents was highest in spring (weekday: males 0.27, females 0.22; weekend: males 0.04, females 0.03 SED/day) and summer seasons (weekday: males 0.46, females 0.36; weekend: males 0.03, females 0.03 SED/day). Median UVR doses were higher on schooldays versus weekend days in all seasons except winter in both genders (spring, *p* < 0.001; summer, *p* < 0.001; autumn, males, *p* = 0.02; females, *p* < 0.001; winter (non-significant)). In comparison to white adolescents, South Asian females gained lower UVR doses (*p* < 0.001) on spring and summer weekends, and South Asian males gained lower UVR doses on summer weekends (*p* < 0.001), with no significant differences on schooldays.

### 3.7. Time Outdoors

**Figure 5:**
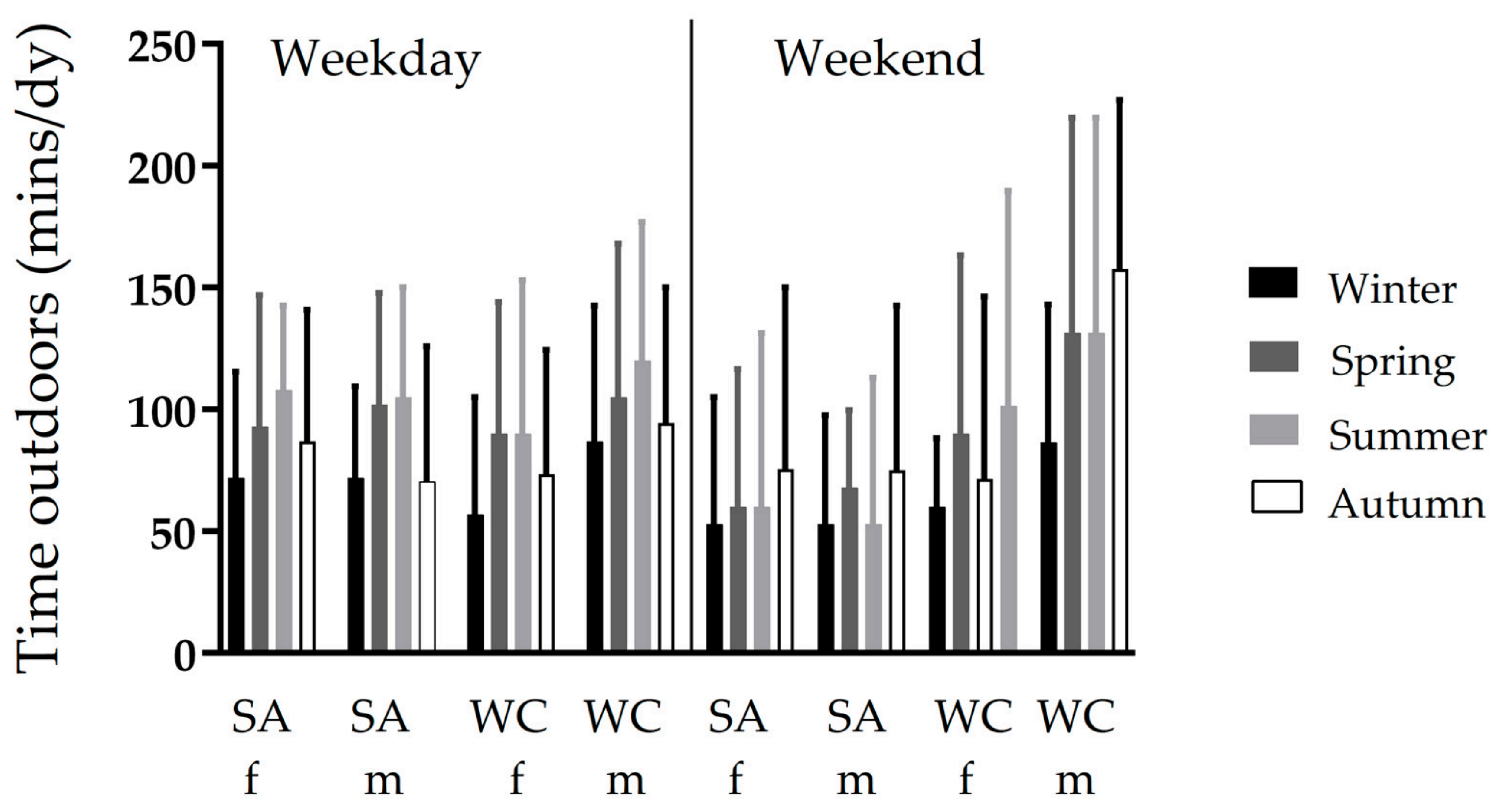
Median (IQR) time outdoors per day (minutes/day) on weekdays and on weekends in each season for the adolescent groups (SA, South Asian; WC, white Caucasian; f, females; and m, males.) Weekdays: SA winter *n* = 98; spring *n* = 103; summer *n* = 88; autumn *n* = 84; WC winter *n* = 91; spring *n* = 110; summer *n* = 96; and autumn *n* = 94; Weekends: SA winter *n* = 96; spring *n* = 102; summer *n* = 88; autumn *n* = 84; WC winter *n* = 88; spring *n* = 106; summer *n* = 92; and autumn *n* = 94.

Sun exposure diaries provided details of time spent outdoors in each season. Time outdoors for both schooldays and weekends for South Asian female and male adolescents, in comparison with white Caucasian adolescents, is shown in Figure 5. During schooldays, the longest period of time spent outdoors was in summer, which is the season with the most daylight hours and highest ambient UVR.

During schooldays, there was no significant difference in median time outdoors between South Asian females and males: winter: 72 (45 to 116) vs. 72 (45 to 110) mins; spring: 93 (57 to 147) vs. 102 (55 to 148) mins; summer: 108 (63 to 143) vs. 105 (51 to 150) mins; and autumn 87 (48 to 141) vs. 71 (38 to 126) mins. These schoolday times outdoors did not differ significantly from those of the white Caucasians. It was notable that at the weekends, the South Asians decreased their median time outdoors, with similar times for females and males: winter: 53 (23 to 105) vs. 53 (15 to 98) mins; spring: 60 (23 to 116) vs. 68 (28 to 99) mins; summer: 60 (30 to 131) vs. 53 (30 to 113) mins; and autumn: 75 (30 to 150) vs. 75 (23 to 143) mins. The differences between schooldays and weekends were statistically significant in females in winter (*p* = 0.03), spring (*p* = 0.02), and summer (*p* < 0.001), and in males in summer (*p* < 0.001). On summer weekends, both female and male South Asian adolescents spent significantly less time outdoors than the male and female white Caucasian adolescents (*p* < 0.001), and on spring and autumn weekends, female South Asians spent less time outdoors than the white male adolescents (*p* = 0.02 and *p* = 0.04, respectively).

### 3.8. Clothing Worn and Skin Surface Area Exposed

**Figure 6:**
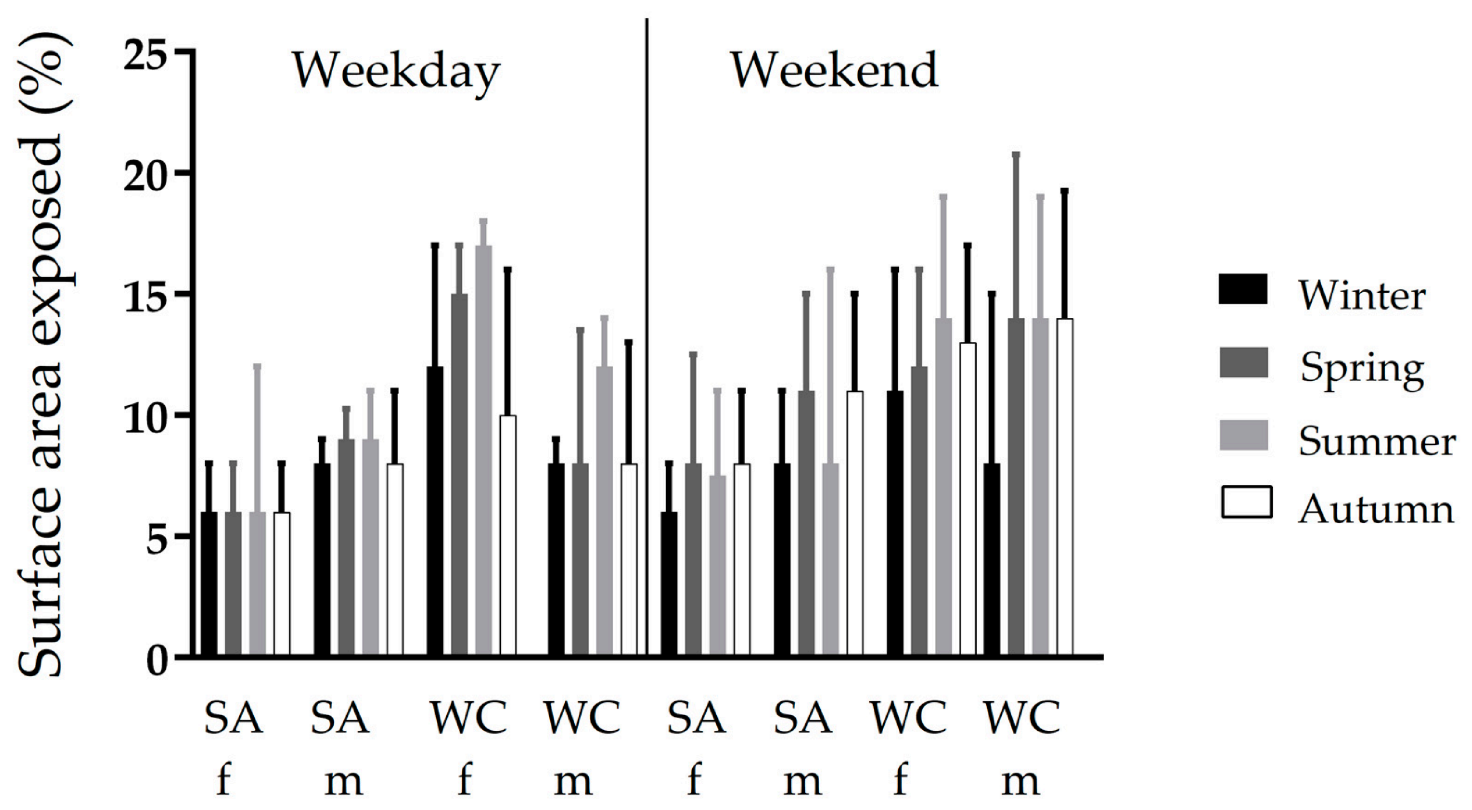
Median (IQR) percentage skin surface area exposed on weekdays and weekends in each season for the adolescent groups (SA, South Asian; WC, white Caucasian; f, females; and m, males). Weekdays: SA winter *n* = 93, spring *n* = 101, summer *n* = 88, and autumn *n* = 81; WC winter *n* = 89, spring *n* = 110, summer *n* = 96, and autumn *n* = 95. Weekends: SA winter *n* = 89, spring *n* = 98, summer *n* = 88, and autumn *n* = 81; WC winter *n* = 84, spring *n* = 107, summer *n* = 93, and autumn *n* = 95.

Skin surface area exposed depended on clothing worn, ranging from shorts and t-shirts (mean 23% of surface area exposed) to long-sleeved tops, trousers/long skirts, and a head garment (only 4% exposed) in South Asians (Figure 6). There was very little variation in the percentage area exposed in different seasons on schooldays, with the South Asian males exposing a mean of 8 to 10% and the females 8 or 9%. During weekends, area exposed increased only moderately with a mean in summer of 10 to 13% in males and 8 to 10% in females.

In comparison, the white female Caucasian adolescents exposed a significantly greater area of skin than the South Asian female adolescents on both schooldays and weekends in all seasons (*p* < 0.001), with the white Caucasian females exposing 15% surface area on summer weekends, which is fifty percent more skin area than the South Asian females. South Asian males exposed the same percentage of skin in all seasons as the white Caucasian males.

### 3.9. Sunscreen Use

Only six South Asian adolescents (four girls, two boys) recorded wearing a dedicated sunscreen product on at least one day in the study period in summer, and a minority of South Asian adolescents wore a facial skin care product incorporating SPF on at least one day in the study period, i.e., 14% in winter and 7% in summer. However, this was still more than the white Caucasian adolescents, none of whom recorded wearing a dedicated sunscreen product in any season, while a small percentage (11% in winter, 6% in summer) of white Caucasian female adolescents wore face cream incorporating SPF.

### 3.10. Sunny Holidays Abroad

There was a large difference in the number of adolescents taking sunny holidays in the two groups: only 28% of South Asian adolescents reported going on holiday during the study year compared to 86% of white Caucasian adolescents (*p* < 0.001).

### 3.11. Predictors of Autumn 25-Hydroxyvitamin D Concentrations

Multiple linear regression analysis of the combined South Asian and white Caucasian data revealed that demographic factors explained 45% (R^2^ = 0.448) of the variation in the end of summer (autumn) 25OHD levels with ethnicity and gender being significant predictors (Table 2). The addition of behavioural factors increased the variation in autumn 25OHD explained by the model (R^2^ = 0.565; Table 2): ethnicity (*p* < 0.001), gender (*p* = 0.005), dietary vitamin D intake (*p* = 0.002), vitamin D supplement intake (*p* < 0.001), and total number of days on sunny holidays (*p* = 0.003) were predictors of 25OHD level.

Males (South Asian and white Caucasian combined) had higher (by 25%) 25OHD levels than females. South Asian adolescents had lower 25OHD (by 45%) levels compared to white Caucasian adolescents. An increase in age was associated with an increase of 1.06 nmol/L in autumn 25OHD level. A 1 µg increase in vitamin D through food intake was associated with an increase in autumn 25OHD of 1.09 nmol/L, and similarly, a 1 µg increase in vitamin D through supplementation was associated with an increase in autumn 25OHD of 1.03 nmol/L. The total number of days on a sunny holiday was a predictor of 25OHD, with a one day increase in holiday being associated with a 1.29 nmol/L increase in autumn 25OHD level.

## 4. Discussion

In this original prospective study, we comprehensively evaluated the influence of sun exposure parameters, as well as oral vitamin D intake, on vitamin D status, with measurements over four consecutive seasons in a cohort of South Asian adolescents living at northerly latitude. Additionally, we compared the findings with those of a study performed using identical protocols at the same location in white Caucasian adolescents [4]. The findings include the extremely high prevalence of year-round severe vitamin D deficiency in South Asian children, particularly in girls; the association with secondary hyperparathyroidism in a high percentage of this group; the contrast in sun exposure behaviour between South Asians and their white counterparts, with the South Asians reducing their sun exposure at weekends compared with schooldays, and spending notably fewer days abroad on sunny holidays; and that while dietary vitamin D intake was low in both groups, it was lower in the South Asian children.

The South Asian adolescents’ median peak circulating 25OHD values were approximately half of those of their white Caucasian counterparts and with less seasonal variation (Figure 1). The modest seasonal rise in circulating 25OHD seen in South Asian girls (winter median 13.2 nmol/L; summer 21.3 nmol/L) was less than in South Asian boys (winter 15.5 nmol/L; summer 33.4 nmol/L). In contrast, white Caucasian adolescents at this latitude reached a 25OHD at summer-end of 61.7 nmol/L, which was associated with a median winter trough above the severe deficiency level, at 36.9 nmol/L [4]. The notable difference in peak 25OHD values in these adolescent groups mirrored those in the UK working-age adult population [22,23,24,25]. The scale of vitamin D deficiency observed in South Asian adolescents is of concern. The majority (79%) demonstrated 25OHD < 25 nmol/L in at least one season, which is far higher than that found in white Caucasian adolescents (16%) [4]. We reported that UK white adolescents with seasonal vitamin D deficiency had significantly lower bone mineral density (BMD) than the standardised age- and sex-matched local population [4], and year-round deficiency, as frequently seen in the South Asian children, may put adolescents at higher risk of low BMD.

We found that low vitamin D status in South Asian adolescents was associated with secondary hyperparathyroidism (defined as serum PTH concentrations >assay reference range) in a high percentage of participants (59% of males and 45% of females in winter; 38% of both males and females during autumn). Previous published data in children are limited. A study based in India of South Asian children with underlying health conditions reported a prevalence of secondary hyperparathyroidism of 20–30% [26]. Consistent with our findings, studies in adult females have found no significant inverse association between PTH and 25OHD concentrations in white Caucasians, whereas significant negative correlations (r = −0.20 to −0.39) have been reported in South Asians, supporting the presence of secondary hyperparathyroidism in the latter group [27,28]. Secondary hyperparathyroidism gives rise to osteoclastic bone resorption, elevating bone turnover markers, and reducing BMD [29]. The high prevalence of vitamin D deficiency and secondary hyperparathyroidism observed in our cohort may compromise their peak bone mass, defined as the maximum BMD attained at the completion of skeletal maturation; this is a critical determinant of lifelong skeletal health [30,31]. The attainment during adolescence and early adulthood is the most important modifiable predictor of future osteoporosis and fractures in both sexes [32].

In light-skinned populations, it is known that 25OHD levels are strongly influenced by sun exposure [33]. An overview of the few earlier studies on sun exposure in adolescents concluded that, in the UK during summer, they spent on average 2 h per weekday outdoors, increasing to approximately 2.5 h on weekends [34,35]. The findings from the current study revealed that South Asian adolescents also spent around 2 h outdoors in summer on schooldays. However, unlike their white counterparts, their outdoor time decreased on weekends. Notably, South Asian boys spent significantly less time outdoors on weekends during the summer (an average of 53 min per day) compared to white Caucasian boys (average of 131 min per day). Furthermore, up to 20% of the South Asian adolescents showed no evidence of venturing outside at all at the weekends. This is in contrast to the more standardised schoolday, where all South Asian boys and almost 95% of girls went outdoors for a length of time.

Data from polysulphone badges showed a seasonal pattern in UVR dose, with peak median values during summer. Median peak UVR doses for South Asian adolescents on schooldays were 0.36 SED/day for females and 0.46 SED/day for males, with significantly lower values at the weekend of 0.03 and 0.03 SED/day, respectively. In their white Caucasian counterparts, the peak was lower in females (0.31 SED/day) but higher in males (0.72 SED/day) on schooldays but higher than South Asian adolescents on weekends, 0.13 SED/day (females) and 0.15 SED/day (males). The lower UVR exposure at weekends relative to weekdays in white Caucasian adolescents did not match their time outdoors data and may reflect compliance issues outside of school, potentially due to outdoor weekend activities such as organised sports where polysulphone badges are not worn, or lifestyle, such as the use of more shaded areas at weekends when outdoors. Polysulphone badge data is scarce and we are unaware of other studies performed in South Asian adolescents. In South Asian adults at the same latitude, we found a higher median UVR dose on weekdays and weekends (0.70 and 0.20 SED/day) than in adolescents (0.38 and 0.03 SED/day) in summer [16]. Occupation is a strong influencer of sunlight exposure [36], and likewise, the structured school schedule incorporating outdoor activities appears to have led to South Asian adolescents accumulating higher SED levels on weekdays.

Holidays in sunny climates were also shown to be important to the vitamin D status of adolescents living at UK latitude. Less than a third (28%) of the South Asian adolescents in our study went on holiday during their 12-month study period, contrasting starkly with the majority (86%) of white Caucasian adolescents, and with the number of days spent at a sunnier latitude shown to be a significant contributor to their 25OHD levels. This builds on data gained in the study of female adults of differing ethnic groups (white Caucasian, Black, and Asian) living in the UK that indicates that vitamin D status is improved by sun exposure during holidays [37,38].

Pertinent to gaining 25OHD through sun exposure is not only time outdoors, but also percentage of skin surface area exposed to sunlight and the application of sunscreens. We found that skin surface area exposed was low relative to white Caucasians in both genders of South Asian adolescents. South Asian males generally wore long trousers with long-sleeved tops during weekdays and occasionally short-sleeved tops in summer. South Asian females varied their clothing very little through the seasons, with 40% of girls wearing a headscarf, long-sleeved tops, and trousers or long skirts on weekdays. In contrast, white Caucasian adolescents exposed more skin in summer, opting to wear short-sleeved-tops and occasionally shorts or short skirts [4]. Despite this, as in the South Asians adolescents, use of products containing sunscreen was scarce.

There was little change in dietary vitamin D over the seasons, with median intakes ranging from 1.21 to 1.33 μg/day in South Asian adolescents. The median intake for South Asian females (1.39 μg/day) was significantly lower than for white Caucasian females (1.97 μg/day), while all other dietary intakes between genders and ethnicities were similar. Low proportions of South Asian (18%) and white Caucasian (23%) adolescents took vitamin D supplements, but interestingly, South Asian adolescents took larger amounts (median 4.14 μg/day) than white Caucasians (1.43 μg/day), suggesting an awareness of higher risk of vitamin D deficiency in this supplemented group. However, overall median oral vitamin D intake was still far lower (Table 2) than the 5 µg/day nutrient intake level recommended by the World Health Organization [39] and the 10 μg/day intake advised by the UK Scientific Advisory Committee on Nutrition [7]. This is similar to findings in South Asian and white adults in the UK [16], and other studies in childhood [40,41], but lower than other studies in adults in other European countries [42,43].

When examining the combined contributions of demographic and behavioural factors on vitamin D status through multiple linear regression, ethnicity was observed to have the strongest influence. The effects of skin pigmentation, and possibly other genetic differences between the ethnic groups, contributed to the South Asian adolescents having on average a 25OHD value 45% lower than the white Caucasian adolescents. These findings are consistent with a study in adults that found Asian ethnicity was associated with a lower 25OHD status (by 31.4 nmol/L) [23]. The other key determinants of levels of 25OHD in our study were gender, number of days on a sunny holiday abroad, and oral vitamin D intake (diet and supplementation). Studies in adults have found a significant influence of dietary vitamin D on 25OHD status [40,44,45,46], and based on our model, total oral vitamin D intake of ~1 ug/day increases peak 25OHD by approximately 1 nmol/L. Moreover, our model indicates that 1 day of sunny holiday (per year) increases peak 25OHD level by 1.29 nmol/L, highlighting the major difference that 1–2 sunny holidays abroad can make to vitamin D status in adolescents living at a northerly latitude.

The strengths of this study include a four-season assessment encompassing personal UVR dose, time outdoors, vitamin D from diet and supplements, and serum 25OHD, giving a comprehensive assessment of lifestyle and behaviour in this under-observed adolescent population. However, there are some limitations to collecting lifestyle data. Dosimeters can be obscured by clothing and it is challenging to be sure of compliance outside of school hours [35]. Diet diaries require an estimate of food amounts consumed and can be subject to bias, with participants selectively recalling more healthy food options [47,48]. A review of national climatological datasets shows that, overall, the weather conditions of 2011 and 2013 were very similar, though with month by month variability (Metoffice.gov.uk) [49]; moreover, year-to-year variation in weather conditions or ambient UV radiation may have influenced the comparison between South Asian and white Caucasian adolescents, even though identical protocols were used. Future research could include evaluation of BMD and musculoskeletal function with respect to vitamin D status in South Asian adolescents.

## 5. Conclusions

In conclusion, this longitudinal study has shown a very high prevalence of severe vitamin D deficiency (25OHD < 25 nmol/L), i.e., affecting 80% of UK South Asian adolescents in at least one season of the year, and frequently occurring year-round, particularly in girls. This was accompanied by secondary hyperparathyroidism in a substantial number of adolescents and may potentially compromise their peak bone mass acquisition. South Asian adolescents had low levels of sunlight exposure on weekend days, despite their lower risk of sunburn than in white adolescents, thus reducing their ability to initiate vitamin D synthesis in the skin. In addition to ethnicity and gender, key influencers of vitamin D status included number of days spent on sunny holidays, which were fewer in South Asian than white adolescents, and total oral intake of vitamin D. Sunlight exposure may have other benefits to health in addition to vitamin D synthesis [50], and our findings support that sun exposure advice should be given in a more individualised manner to better accommodate children of different skin types. Additionally, while oral vitamin D supplements are advised for darker skin people residing in the UK [7], compliance is low and effective communication of this guidance is required for South Asian adolescents.

## Figures and Tables

**Table 1:** Characteristics of South Asian and white Caucasian adolescents.

Characteristic	South Asian Adolescents (*n* = 138)	White Caucasian Adolescents (*n* = 131)
Age, years	13.5 (0.99)	13.5 (0.8)
BMI, kg/m^2^	22.5 * (4.4)	21.1 ** (3.2)
Gender		
Male	63 (46)	51 (39)
Female	75 (54)	80 (61)
Skin type	V, *n* = 138	I, *n* = 15
		II, *n* = 38
		III, *n* = 62
		IV, *n* = 16

Data are mean (SD) for age and BMI, and number (percentage) for gender. * *n* = 122, ** *n* = 116.

**Table 2:** Multifactorial regressions of autumn 25OHD on demographic and behavioural factors.

Factor	Estimate (95% CI)	*p* Value	Change in R^2^
Demographic factors alone	(*n* = 220, R^2^ = 0.448)		
Age	1.07 (0.99 to 1.15)	0.088	
Sex			
Female	1.00		
Male	1.21 (1.05 to 1.38)	0.006	
Log (BMI)	0.73 (0.49 to 1.08)	0.120	
Ethnicity			
White Caucasian	1.00		
South Asian	0.44 (0.39 to 0.49)	<0.001	
Demographic and behavioural factors	(*n* = 168, R^2^ = 0.565)		
Age, years	1.06 (0.99 to 1.15)	0.109	
Sex		0.005	
Female	1.00		
Male	1.25 (1.07 to 1.47)		
Log (BMI)	0.73 (0.49 to 1.10)	0.128	
Ethnicity		<0.001	0.120
White	1.00		
South Asian	0.55 (0.46 to 0.66)		
Dietary vitamin D intake (μg/day)	1.09 (1.03 to 1.16)	0.002	0.026
Vitamin D supplement intake (μg/day)	1.03 (1.02 to 1.05)	<0.001	0.044
Time outdoors (h/day, weekend)	1.00 (1.00 to 1.00)	0.638	0.001
Time outdoors (h/day, weekdays)	1.00 (0.99 to 1.01)	0.530	0.001
UVR dose (Log[SED/day], weekend)	1.00 (0.88 to 1.05)	0.822	0.003
UVR dose (Log[SED/day], weekdays)	0.96 (0.89 to 1.06)	0.402	0.002
Surface area exposed (Log[%/day], weekend)	1.27 (0.95 to 1.04)	0.840	0.005
Surface area exposed (Log[%/day], weekdays)	0.97 (0.74 to 1.28)	0.074	0.009
Holiday	1.29 (1.09 to 1.53)	0.003	0.034
Intercept	64.72 (14.73 to 281.46)	<0.001	

BMI, body mass index; SED, standard erythemal dose; UVR, ultraviolet radiation; and CI, confidence interval.

## Data Availability

The original contributions presented in this study are included in the article. Further inquiries can be directed to the corresponding author.
